# Production of Selenomethionine-Enriched *Bifidobacterium bifidum* BGN4 via Sodium Selenite Biocatalysis

**DOI:** 10.3390/molecules23112860

**Published:** 2018-11-02

**Authors:** Weihong Jin, Cheolho Yoon, Tony V. Johnston, Seockmo Ku, Geun Eog Ji

**Affiliations:** 1 Department of Food and Nutrition, Research Institute of Human Ecology, Seoul National University, Seoul 08826, Korea; kimweihong@hanmail.net; 2 Korea Basic Science Institute, 145 Anamro, Sungbuk-Gu, Seoul 02841, Korea; chyoon@kbsi.re.kr; 3 Fermentation Science Program, School of Agriculture, College of Basic and Applied Sciences, Middle Tennessee State University, Murfreesboro, TN 37132, USA; tony.johnston@mtsu.edu; 4 Research Center, BIFIDO Co. Ltd., Hongcheon 25117, Korea

**Keywords:** *Bifidobacterium*, functional foods, food additives, feed additives, probiotics, organic selenium, inorganic selenium, selenomethionine

## Abstract

Selenium is a trace element essential for human health that has received considerable attention due to its nutritional value. Selenium’s bioactivity and toxicity are closely related to its chemical form, and several studies have suggested that the organic form of selenium (i.e., selenomethionine) is more bioavailable and less toxic than its inorganic form (i.e., sodium selenite). Probiotics, especially *Bifidobacteriium* and *Lactobacillus* spp., have received increasing attention in recent years, due to their intestinal microbial balancing effects and nutraceutical benefits. Recently, the bioconversion (a.k.a biotransformation) of various bioactive molecules (e.g., minerals, primary and secondary metabolites) using probiotics has been investigated to improve substrate biofunctional properties. However, there have been few reports of inorganic selenium conversion into its organic form using *Bifidobacterium* and *Lactobacillus* spp. Here we report that the biosynthesis of organic selenium was accomplished using the whole cell bioconversion of sodium selenite under controlled *Bifidobacterium bifidum* BGN4 culture conditions. The total amount of organic and inorganic selenium was quantified using an inductively coupled plasma-atomic emission spectrometer (ICP-AES). The selenium species were separated via anion-exchange chromatography and analyzed with inductively coupled plasma-mass spectrometry (ICP-MS). Our findings indicated that the maximum level of organic selenium was 207.5 µg/g in selenium-enriched *B. bifidum* BGN4. Selenomethionine was the main organic selenium in selenium-enriched *B. bifidum* BGN4 (169.6 µg/g). Considering that *B. bifidum* BGN4 is a commercial probiotic strain used in the functional food industry with clinically proven beneficial effects, selenium-enriched *B. bifidum* BGN4 has the potential to provide dual healthy functions as a daily supplement of selenium and regulator of intestinal bacteria. This is the first report on the production of organic selenium using *B. bifidum* spp.

## 1. Introduction

Selenium is a micronutrient essential to the maintenance of human health that plays an important role in human diseases, including cancer and cardiovascular diseases [1,2,3]. Selenium deficiency can cause Keshan disease and Kashin–Beck disease, both of which have high fatality rates [4,5]. Wei et al. [6] demonstrated that dietary selenium intake was useful for the suppression of Type 2 diabetes, both for onset and development. The US recommended daily allowance (RDA) of selenium is 55 µg (0.7 µ mol)/day [7]. According to the European Food Safety Authority (EFSA) [8], sodium selenite is the most common inorganic form of elemental selenium for human and animal use. However, selenium’s bioactivity and toxicity are closely related to the form in which it exists, and several studies have suggested that the organic form of selenium is more bioavailable and less toxic than its inorganic form [9,10].

According to a recent market research report [11], the U.S. probiotics market can be divided into three categories: (i) probiotic food and beverages, (ii) probiotic dietary supplements, and (iii) animal feed probiotics. Among them, the food and beverage sector demonstrated the largest growth in 2015 and accounted for more than 85% (2.5 billion USD) of the total U.S. probiotic microorganism market sales. The remaining 10% and 5% of the market was dominated by dietary supplements and animal feed additives, respectively. Another industry report forecasts the global probiotics market will grow at a compound annual growth rate (CAGR) of 7.01% from 2017–2021 [12]. Therefore, the major players in the food and nutraceutical industries are investing in research and business development (R&BD) to diversify their product categories, gain competitive advantage, and develop differentiated probiotic bacteria to meet consumers’ desires [13]. Among the various probiotic species, *Bifidobacteriium* and *Lactobacillus* spp. are commonly found in the naturally-occurring microbiota of healthy breast-fed infants’ intestinal tracks. *Bifidobacteriium* and *Lactobacillus* have received increased attention in recent years as probiotics, due to their intestinal microbial balancing effects and medical benefits, including anti-inflammatory effects, alleviation of lactose intolerance, relief of constipation, anticholesterolaemic effects, anticancer activity, and tolerogenic immune responses to their hosts (human and animals) [13,14,15,16].

**Figure 1:**
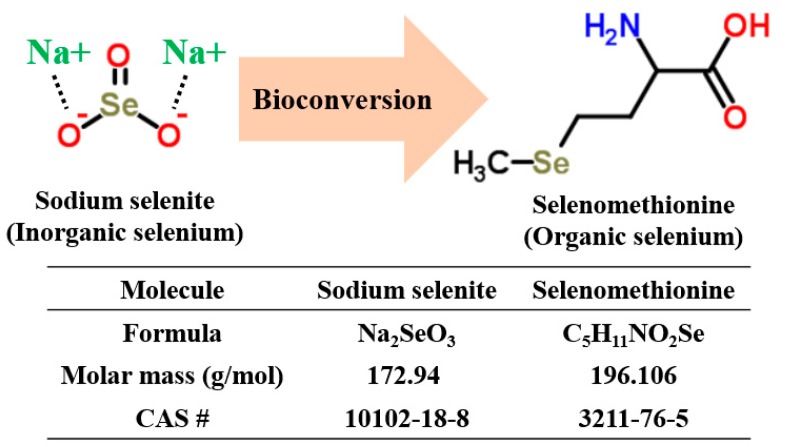
Bioconversion of inorganic selenium (sodium selenite) using microorganisms responsible for organic selenium (selenomethionine) production.

According to recent studies, some yeast strains (*Saccharomyces cerevisiae* and *S. bayanus*) [17,18] and mushrooms (*Lentinus edodes*) [19] have demonstrated the ability to take up inorganic selenium in culture media and transform inorganic selenium into its organic form via a bioconversion process (Figure 1).

Using these selected microorganisms, certain organic selenium-enriched foods and feeds (e.g., selenium-enriched yeast cell biomass, wine and shiitake mushrooms) were developed. Therefore, multiple groups and scholars applied inorganic selenium into probiotics culture media as a media ingredient to produce organic selenium-enriched probiotics or fermented food; however, these studies have shown the limitations of prepared organic selenium-enriched probiotic bacteria due to low organic selenium yields and lethal effects of inorganic selenium on bacterial cell growth [20,21,22]. Accordingly, the harvested cell mass and organic selenium concentrations in the selenium-enriched bacteria were not satisfactory. Hence, the effective production of probiotic bacteria and their functional biogenic metabolites in a reproducible manner is required to achieve high biomass, high organic selenium yields, and reduced operational costs. The objectives of this project are to: (i) identify the probiotic strains resistant to inorganic selenium present in the culture medium; (ii) evaluate the influence of high sodium selenite levels on selected microbial growth; (iii) counteract the reduced cell mass due to poor selenium tolerance by probiotic strains using a controlled culture condition; and (iv) evaluate the selenium uptake efficiencies and biocatalytic properties of promising strain(s) capable of providing dual health functions (as both a selenium supplement and intestinal bacteria regulator).

## 2. Results and Discussions

### 2.1. Subsection Cell Screening and the Effect of the Cultivation Conditions on the Bacterial Cell Mass

Whole-cell biocatalysts are widely used to efficiently biosynthesize value-added products by increasing the bioavailability with bioconversion of existing bioactive materials [23,24,25,26]. According to Mrvčić et al. [27], whole cells of certain lactic acid bacteria (LAB) species can assimilate metalloid ions in culture media and accumulate the metalloproteins intracellularly via two basal mechanisms: biosorption and bioaccumulation. Generally, the low level of sodium selenite has enhanced the microbial growth rates and amino acids levels of *Lactobacillus* spp.; however, a high level of selenium inhibited the bacterial growth and triggered biological detoxification by converting sodium selenite to elemental selenium, which had significantly accumulated in the microorganism periphery [21,22]. Thus, organic selenium is obtained either from the surface binding of elemental or inorganic selenium [28]. After the inorganic selenium is deposited within the microorganism, the molecule is converted into organic selenium by the microorganism. Thus, the yield of organic selenium may increase proportionally with the amount of cell biomass produced. For the preliminary screening process, nine LAB strains were cultured in 15 mL of modified de Man, Rogosa and Sharpe (MRS) media containing with a high level of sodium selenite (1 mM, 172.9 mg/L) for 18 h. When cultured in the modified MRS media containing sodium selenite (mMRS-SS), microorganisms express a blood or dark red color or maroon hue. The culture medium turned red during the incubation following the addition of sodium selenite. According to Xia et al. [22], the red color of the medium was the result of the formation of non-toxic elemental selenium after the experimental cells were treated with a high selenium concentration (>4 mg/L).

After cell cultivation, washing and freeze-drying processes were carried out to obtain pure and dried cell biomass. Among the nine strains of LAB, four strains of *Lactobacillus* (*L. reuteri* KCTC 53608, *L. cremoris* ATCC 19257, *L. plantarum*, and *L. brevis* GABA100) were inhibited by the presence of 1 mM of sodium selenite in the media. The level of recovered cell biomass of *L. reuteri* KCTC 53608, *L. cremoris* ATCC 19257, *L. plantarum*, and *L. brevis* GABA100 were 3.5 ± 0.3, 3.7 ± 0.3, 1.1 ± 0.3, and 4.4 ± 0.3 mg, respectively (*n* = 3). The four *Lactobacillus* strains with low biomass yields (<5 mg of recovered cell biomass) were excluded from subsequent experiments. The five LAB strains that showed resistance to sodium selenite (*L. bulgaricus* KCTC 3188, *L. acidophilus* KCTC 3142, *L. casei* KFRI 704, *L. brevis* 353, and *B. bifidum* BGN4) were used to evaluate cell growth under various selenium treatment conditions to quantify the total selenium content of cells and to evaluate the selenium species produced.

To reduce the toxicity of sodium selenite, the cultivation conditions were divided into two phases: (i) the microbial growth and proliferation phase, during which the probiotic microorganisms were proliferated to the required levels; and (ii) the organic selenium production and accumulation phase, during which the sodium selenite stock solution was fed to the cell culture. We used a two-step fed-batch technique in which the substrate is treated with the microbial medium over time to prevent the substrate from adversely affecting microbial growth. Specifically, two-step fed-batch cultivation methods are considered a cultivation option for simple systems in which the microbial media ingredients and/or substrates are toxic to the beginning of a batch process [29]. These culture methods have been utilized to achieve high levels of microbial production and/or animal cell replication as a result of high cell biomass recovery, over a relatively large span of time, under the toxic conditions [30].

*L. bulgaricus* KCTC 3188, *L. acidophilus* KCTC 3142, *L. casei* KFRI 704, *L. brevis* 353, and *B. bifidum* BGN4 were cultured under various culture conditions and the recovered cell biomasses under these conditions were compared to the biomass produced in common MRS. Table 1 shows the changed dry weights of the selenium-enriched LAB biomass (*n* = 3) as a function of sodium selenite addition into the cell growth medium.

The addition of sodium selenite at 12 h did not lead to a statistically significant reduction of the cell mass (*n* = 3, *p* > 0.01). On the other hand, the addition of sodium selenite at 0 h significantly inhibited the growth of *L. bulgaricus* KCTC 3188, *L. acidophilus* KCTC 3142, *L. casei* KFRI 704, *L. brevis* 353, and *B. bifidum* BGN4 by 40, 26.9, 41.9, 31.1, and 75.9%, respectively, compared to the control group (*n* = 3, *p* < 0.01). Selenium compounds inhibited the growth of *Staphylococcus aureus*, *Lactobacillus*, and *Bifidobacterium*. [21,31,32].

According to Diowksz et al. [33], cell growth inhibition of three LAB (*L. casei*, *L. brevis*, and *L. sanfrancisco*) was demonstrated with ≥1 mg/L of selenium dioxide. Andreoni et al. [34] reported that a concentration of sodium selenite exceeding 1 mg/L inhibited the growth of *L. paracasei*, *L. kefir*, and *L. rhamnosus*. Despite the fact that we added 172.9 mg/L of sodium selenite to the culture media in this study, levels of selenium-enriched LAB biomass were not notably decreased, as compared to the control group. Since the addition of selenium 12 h after incubation did not lead to a reduction of the final probiotic cell mass, the present preliminary method offers an effective solution for overcoming the reduction of bacterial cell mass caused by poor microbial selenium tolerance.

### 2.2. Quantification and Qualification of Organic Selenium in Microorganisms

Based on the previously mentioned data, sodium selenite was added to the MRS media after 12 h incubation and the total amount of organic selenium in the harvested cells was quantified. Endopeptidase hydrolysis was conducted to degrade the cell structure prior to the selenium species analysis. Endopeptidase has been used to break down the peptide bonds of selenium-containing proteins in selenium enriched microorganisms. This method is also considered the most effective pre-treatment of selenium species [35,36]. Table 2 shows total organic selenium content of selenium-enriched LAB quantified using inductively coupled plasma-atomic emission spectrometer (ICP-AES).

**Figure 2:**
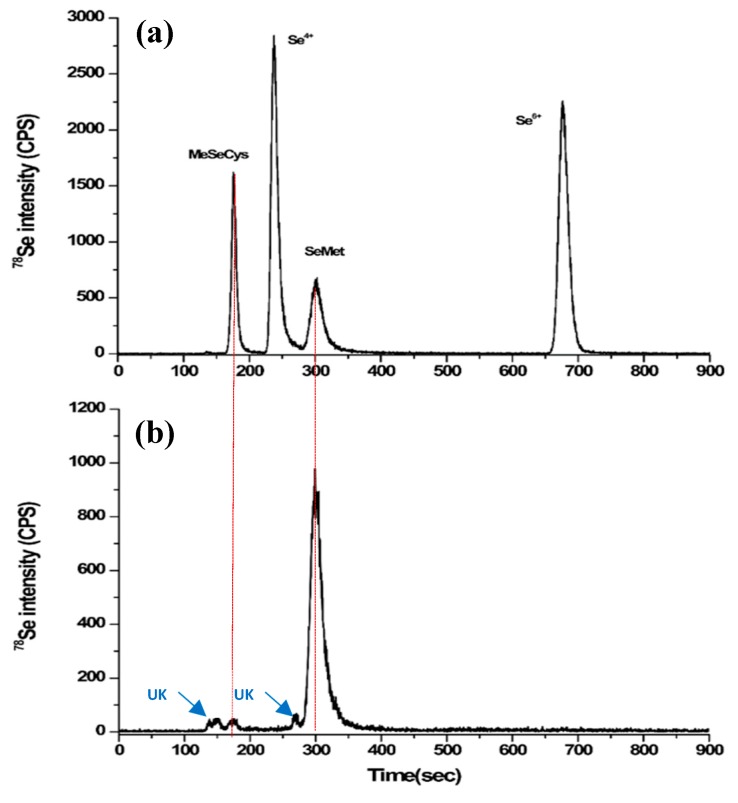
The cromatographic profiles of a standard selenium mixture (**a**) and enzymatically-extracted organic selenium from selenium-enriched *B. bifidum* BGN4 (**b**) analyzed by HPLC-ICP-MS. UK denotes unknown.

*L. casei* KFRI 704 showed the lowest organic selenium concentration (35 ± 0.2 µg/g, *n* = 3) and *B. bifidum* BGN4 produced the highest concentration of total organic selenium (207.51 ± 25 µg/g, *n* = 3). The total organic selenium production of *B. bifidum* BGN4 was significantly higher (*n* = 3, *p* < 0.01) when sodium selenium was added to the culture after 12 h, although the yield of *B. bifidum* BGN4 biomass was significantly low compared to the other four strains (*n* = 3, *p* < 0.01). Multiple researchers and research groups have shown that when inorganic selenium (sodium selenite and selenium dioxide) is supplied to a cell culture media, selenium molecules accumulate in the cellular membrane and/or intracellular region of LAB in the form of selenoproteins or elemental selenium. In liquid media, dissolved sodium selenite is generally present as selenite (Se^+4^) and/or selenate (Se^+6^). According to Xia et al. [21] and Diowksz et al. [33] *L. plantarum* and *L. bulgaricus* deposit elemental selenium via cell detoxification. In Figure 2a, a typical HPLC-ICP-MS chromatogram of selenium standards is shown with the following elution times (sec): methylselenocysteine, tret = 170–180; selenite, tret = 230–240; selenomethionine, tret = 300–310; and selenate, tret = 670–680. The ICP-MS chromatogram in Figure 2b shows the chromatographic profiles of enzymatically-extracted organic selenium from selenium-enriched B. *bifidum* BGN4 analyzed via HPLC-ICP-MS. As shown in Figure 2b, the major species of selenium-enriched *B. bifidum* BGN4 was selenomethionine (169.6 µg/g).

Low levels of methylselenocysteine and two other unknown substances were identified. However, neither selenite (Se^4+^) nor selenate (Se^6+^) were detected in the sample. According to Yin et al., [37], *B. longum* NQ-1501 cultured in trypticase-phytone-yeast extract (TPY) media containing sodium selenite produced a total of 82 μg/g organic selenium. In their work, <60 μg/g (about 60%) of organic selenium was selenomethionine. Multiple research groups have developed selenium-enriched fermented food products. Alzate [38] reported methylselenocysteine- and selenocysteine-enriched yogurt containing 0.7 μg/g of total selenium. Penas et al. [39] and Bryszewska et al. [40,41] also reported selenomethylcysteine- (1.29 μg/g) and selenomethionine- enriched (3.56 μg/g) sauerkraut and bread, respectively.

A previous study suggested that the relative bioavailability of selenomethionine compared to sodium selenite is 147% for liver glutathione peroxidase activity and 336% for weight gain in channel catfish, which indicates that the selenium requirement in the diets of channel catfish is reduced by selenomethionine more so than by inorganic selenium [42]. Claire et al. [43] suggested that selenomethionine inhibited the growth of human tumor cell lines (MCF-7/S breast carcinoma, DU-145 prostate cancer cells, and UACC-375 melanoma) over the range of 45–130 µM. The growth inhibition of normal diploid fibroblasts required 1 mM selenomethionine, much higher than the requirements of cancer cell lines. The bioavailability and low toxicity of selenomethionine make it an appropriate supplemental form of selenium for humans and animals [44]. According to the Toxnet of the National Institutes of Health (NIH), selenomethionine is about 40 times less toxic than sodium selenite as measured by laboratory animal toxicity. [45]. Specifically, the median lethal dose (LD50) of selenium forms are as follows: 7 mg/kg for sodium selenite [46] and 25.6 mg/kg for selenomethionine [47]. The minimum selenium requirement for the prevention of Keshan disease is 20 µg/day. The physiological requirement for maximal glutathione peroxidase (GPx) and selenoprotein P is estimated to be 45–50 µg/day and 30 µg/day for iodothyronine 5 deiodinases (IDIs). In addition, protection against some cancers, such as lung and prostate, requires 120 µg/day [48].

Considering that the US RDA of selenium is 55 µg /day [7] and the amount of selenomethionine produced by *B. bifidum* BGN4 is 169.6 μg/g, *B. bifidum* BGN4 could be used under the growth conditions described herein to produce sufficient selenomethionine levels for commercial biofunctional applications. *B. bifidum* BGN4 is considered one of the most promising probiotics, given its clinically proven benefits and widespread commercial use [13]. Research has suggested that the oral feeding of *B. bifidum* BGN4 can prevent T cell-mediated inflammatory bowel disease and eczema in infants via immunomodulatory effects [49,50]. Multiple in vivo and in vitro experiments using *B. bifidum* BGN4 showed its strong cell adhesion properties, anti-carcinogenic effects on cell lines, cell safety and suppressed allergic responses in mouse models [13,15,51]. Given the low toxicity of elemental selenium, the high bioactivity of selenomethionine, and the high selenium uptake efficiency of *B. bifidum* BGN4 shown in the current study, selenium-enriched *B. bifidum* BGN4 may be an excellent vehicle for supplementation of dietary selenium and regulation of intestinal bacteria.

## 3. Materials and Methods

### 3.1. Microbial Culture Condition and Initial Cell Screening

Three replicate pre-cultures of nine LAB (*Lactobacillus reuteri* KCTC 53608, Lactobacillus bulgaricus KCTC 3188, *Lactobacillus* acidophilus KCTC 3142, *Lactobacillus* casei KFRI 704, *Lactobacillus* cremoris ATCC 19257, *Lactobacillus* plantarum, *Lactobacillus* brevis 353, *Lactobacillus* brevis GABA100, and Bifidobacterium bifidum BGN4) were created by inoculating at 1% (*v*/*v*) from a glycerol frozen stock (−80 °C) in MRS media and stored for 18 h under anaerobic conditions at 37 °C prior to use. All cell strains were obtained from BIFIDO Co., Ltd. (Hongchen, Korea). The activated microorganisms were inoculated in a commercially-available MRS medium (DifcoTM & BBLTM, IL, USA) and modified MRS media containing sodium selenite, mMRS-SS. A sodium selenite (Na_2_O_3_Se, Sigma-Aldrich, St. Louis, MO, USA) stock solution (172.9 mg dissolved in 10 mL of DW) was prepared and filtered using a 0.2 µm syringe filter. Fifteen µL of this stock solution was added to the MRS culture medium (15 mL) to produce mMRS-SS. The final sodium selenite concentration in the culture medium was adjusted to 1 mM. The initial pH of the culture broths was adjusted to 7.0 via 1 N NaOH at 25 °C. All culture media were supplemented with 0.05% (*w*/*v*) l-cysteine hydrochloride to maintain anaerobic microbial culture conditions. Naturally-occurring microbiota from the MRS and mMRS-SS media were removed using a 0.2 µm-cutoff syringe microfilter membrane (Stericup^®^-GV Filter Units, Burlington, MA, USA). Each medium was inoculated with 1% (*v*/*v*) activated-inoculum culture and then anaerobically cultured at 37 °C for 18 h in a water bath (150 rpm). Of the nine LAB strains, five cell strains (*L. bulgaricus* KCTC 3188, *L. acidophilus* KCTC 3142, *L. casei* KFRI 704, *L. brevis* 353, and *B. bifidum* BGN4) showed resistance to sodium selenite via the initial cell screening process. The cell biomasses were harvested by centrifugation (139× *g*, 20 min) and washed twice with deionized water (DI) before being freeze-dried. The biomass from each culture was weighed after freeze-drying.

### 3.2. Selection of Selenium-Enriched Probiotics

The ultimate goal of this study was to stimulate microorganisms to produce organic selenium from inorganic selenium, so it was imperative to measure the toxicity of inorganic selenium to probiotic cells. Fed batch processing is a commonly used culture technique to reduce the toxicity of certain media components and produce large quantities of secondary metabolites from microorganisms. Two-step fed-batch fermentations were conducted for the effective production of cell biomass in this study. The first cultivation step was to increase the total probiotic cell biomass via conventional MRS feeding [29,30]. The second step was designed to minimize the toxicity of sodium selenite and increase organic selenium productivity by supplementing sodium selenite to the MRS media. The five selected strains of cells were cultivated in MRS media for 12 h, after which 1 mM of sodium selenite was added as an inorganic selenium substrate to the media. All cell samples were cultured in a shaking water bath (37 °C, 150 rpm) for an additional 36 h (a total of 2 days). The selenium-enriched microbial samples were harvested by centrifugation (139× *g*, 20 min), washed twice with DI, and freeze dried. Biomass weights were measured as previously described.

### 3.3. Quantification of Total Selenium, Organic Selenium, and Selenium Species

Enzymatic hydrolysis using endopeptidase has been proven to be the most effective method for the quantification of organic selenium species without altering their oxidation states [52]. Therefore, proteolytic treatments were conducted in this study for the quantification of total selenium and total organic selenium. For the extraction of the total organic selenium compounds and assessment of the selenium species, the selenium-enriched LAB powder harvested from 15 mL of culture was dissolved in 5 mL of phosphate buffer containing 20 mg of pronase E from Streptomyces griseus (metalloendopeptidases, 4,000,000 PU/g, EC 3.4.24.4) and incubated at 37 °C for 16 h [35,36]. The samples were then filtered through a sterile filter (0.45 µm, Pall Corporation, Port Washington, NY, USA). Total organic selenium was quantified using an inductively coupled plasma-atomic emission spectrometer (ICP-AES, Optima-4300 DV, Perkin Elmer, Norwalk, CT, USA). Selenium species determination was performed using an inductively coupled plasma mass spectrometer (ICP-MS, Agilent 7700, Agilent Technologies, Tokyo, Japan) equipped with an octopole collision cell. Chromatographic separations were performed using a Model 1260 HPLC pump (Agilent, Wilmington, DE, USA) as the delivery system. The selenium species were separated using a Hamilton (Reno, NV, USA) PRP X-100 anion exchange column (250 mm × 4.1 mm id, 10 μm particles) at 40 °C. The flow rate was maintained at 1 mL/min, and the injection volume was 20 μL. The mobile phase consisted of 2 mM ammonium citrate in 2% methanol (Solution A) and 10 mM ammonium citrate in 2% methanol (Solution B). The gradient elution began with 80% solution A and 20% solution B and was adjusted as follows: A from 80% to 0%, 2–10 min; and A from 90% to 82%, 10–18 min. The ICP-MS operating conditions were as follows: forward power of 1550 W; plasma gas flow rate of 10.5 L/mL; carrier gas flow rate of 0.8 L/mL; makeup gas flow rate of 0.30 L/min; sample depth of 8.0 mm; collision gas (He gas) flow of 3.5 mL/min; quadrupole bias of −16 V; octopole bias of −18 V; and isotopes monitoring at 77Se and 78Se. Seleno-DL-methionine (C5H11NO2Se) and se-(methyl) selenocysteine hydrochloride (C4H9NO2Se·HCl) were used as the HPLC-ICP-MS standards. Enzyme pronase E (protease XIV type) was purchased from Sigma-Aldrich (St. Louis, MO, USA). A phosphate buffered saline (PBS) was prepared by dissolving 8 g NaCl, 0.2 g KCl, 1.44 g Na_2_HPO_4_, and 7.4 g KH_2_PO_4_ in 1 L of DI with a final pH of 7.4. The buffer was autoclaved at 121 °C for 15 min prior to use.

### 3.4. Statistical Analysis

Experiments were performed in triplicate, and the data were shown as mean ± standard deviation. A t-test and one-way analysis of variance (ANOVA) with Duncan post-hoc test (*p* < 0.01) were performed with the statistics package GNU R, version 3.1.2 [30].

## 4. Conclusions

Selenium is a trace element that is essential to human health and has received considerable attention due to its nutritional value. Various microorganism strains were screened to select the strains with inorganic selenium uptake properties and the ability to transform inorganic selenium to the organic form. However, due to the microorganisms’ poor inorganic selenium tolerance, the growth of selenium-enriched cells was inhibited by a small quantity of sodium selenite. To solve the poor selenium tolerance of lactic acid bacteria and identify a strain with both high selenium uptake efficiency and the ability to transform inorganic selenium into an organic form, this experiment utilized nine strains of edible lactic acid bacteria. The total amount of selenium was quantified using an inductively coupled plasma-atomic emission spectrometer (ICP-AES). The selenium species were separated via anion-exchange chromatography and analyzed using inductively coupled plasma-mass spectrometry (ICP-MS). The reduction of bacterial cell mass due to inorganic selenium toxicity was solved by an adjustment of the time of selenium addition (12 h after incubation), and *B. bifidum* BGN4 was found to be the most potent selenium-enriched strain. The adjusted selenium addition time enhanced the cell mass of the selenium-enriched *B. bifidum* BGN4 by overcoming the microorganisms’ poor selenium tolerance. Chromatography results showed that selenomethionine (169.6 μg/g) was the main organic selenium form in selenium-enriched *B. bifidum* BGN4. Considering that *B. bifidum* BGN4 is a well-known probiotic strain with clinically-proven benefits, selenium-enriched *B. bifidum* BGN4 may provide dual healthy functions, both as a daily selenium supplement and an intestinal bacteria regulator.

## Figures and Tables

**Table 1:** Dry weight of selenium-enriched microorganism grown in de Man, Rogosa and Sharpe (MRS) containing 1 mM (172.9 mg/L) of sodium selenite. Data is shown as the mean ± SD of the triplicate experiments.

Cell Strains	Dry Weight of Selenium-Enriched LAB (mg)		
	SS ^1^ Added at 0 h	SS Added at 12 h	Control (No SS Added)
*L. bulgaricus* KCTC 3188	15 ± 0.62 **	24.3 ± 0.55	25 ± 0.38
*L. acidophilus* KCTC 3142	16.3 ± 0.40 **	22.8 ± 0.42	22.3 ± 0.26
*L. casei* KFRI 704	15 ± 0.49 **	25.6 ± 0.31	25.8 ± 0.56
*L. brevis* 353	21 ± 0.49 **	31.1 ± 0.55	30.5 ± 0.81
*B. bifidum* BGN4	5.3 ± 0.50 **	20.9 ± 0.53	22 ± 0.35

^1^ SS denotes Sodium Selenite. ** Values are significantly different compared to the control group (*p* < 0.01).

**Table 2:** Total Organic Selenium in Selenium-Enriched Microorganisms Quantified using inductively coupled plasma-atomic emission spectrometer (ICP-AES). Data is shown as the mean ± SD of the triplicate experiments.

Cell Strains	Total Organic Selenium Content of LAB (µg/g)
*L. bulgaricus* KCTC 3188	111.7 ± 0.16 ^a^
*L. acidophilus* KCTC 3142	134.4 ± 1.18 ^b^
*L. casei* KFRI 704	35 ± 0.20 ^c^
*L. brevis* 353	111.8 ± 0.86 ^a^
*B. bifidum* BGN4	207.5 ± 1.25 ^d^

^a,b,c,d^ Values of different superscripts within the same columns indicate significant differences.
